# Dr. Arnott

**Published:** 1857-03

**Authors:** 


☞ Dr. Arnott, of London, has written several papers of late, to show that the mortality following operations, has greatly increased since the introduction of anæsthetics. The agitation of this subject has led to careful statistical enquiry, and we have now a promise of the results in the principal hospitals of the Metropolis, for the past eight or nine years. We await the publication of these with anxiety. Dr. Snow, who from the first has been extensively engaged in the administration of chloroform and ether, has recently been using the vapor of amylene, which is reported to have caused an entire absence of pain. It produced some mental excitement and muscular rigidity in two of the cases. Amylene is made by distilling fusel oil with chloride of zinc. Its vapor is less pungent than that of chloroform.—*Western Lancet.*

